# Development and Application of an Infectious Disease Risk Assessment Framework for Mass Gatherings in the Republic of Korea

**DOI:** 10.56786/PHWR.2026.19.40.3

**Published:** 2026-10-22

**Authors:** Jeonghyeon Oh, Shin Young Park, Youngsu Eom, HyeJeong Kang, Moo Sool Oh, Jeongsu Mun, Jemma Park, Eun-young Kim, Seongsun Kim

**Affiliations:** 1 Division of Epidemiological Data Analysis, Department of Data Science, Korea Disease Control and Prevention Agency Cheongju Korea; 2 Jecheon City Public Health Center Jecheon Korea; 3 Jeonnam-Gwangju Special Metropolitan City, Jeonnam-Gwangju Korea; 4 Division of Infectious Disease Control & Response, Chungcheong Regional Center for Disease Control and Prevention, Korea Disease Control and Prevention Agency Daejeon Korea; 5 Division of Infectious Disease Control & Response, Honam Regional Center for Disease Control and Prevention, Korea Disease Control and Prevention Agency, Jeonnam-Gwangju Korea

**Keywords:** Mass Gatherings, Infectious Disease Risk Assessment, Infectious Disease Risk Assessment Tool, Communicable Disease Control

## Abstract

**Objectives:**

This study outlines the development of the Korea Disease Control and Prevention Agency (KDCA) infectious disease risk assessment framework for mass gatherings, highlights its key components, and examines its application to domestic mass gathering events.

**Methods:**

Government-funded policy research reports, the risk assessment manual, and documentation from exemplary mass gathering risk assessments were reviewed to analyze the framework’s development, key components, and application outcomes.

**Results:**

The KDCA developed an infectious disease risk assessment tool for mass gatherings through a government-funded research project in 2024. This tool was subsequently integrated into standard operating procedures for preparedness and response to establish a national infectious disease risk assessment framework. Beginning in 2025, the framework has been applied to domestic mass gathering events. An analysis of two events involving iterative risk assessments demonstrated that deficiencies identified during the initial assessment were successfully addressed by strengthening education, hygiene and ventilation management, medical and response systems, and risk communication. These targeted interventions reduced the overall risk level to low or very low in the final assessment.

**Conclusions:**

The Korean infectious disease risk assessment framework for mass gatherings provides a standardized approach for systematically assessing infectious disease risks and preparedness, identifying areas for improvement during event planning, and informing preparedness and response measures. Further application across diverse types of mass gatherings and evaluation of the association between assessment results and actual infectious disease events or response outcomes are warranted.

**Key messages**

**① What is known previously?**

Mass gatherings elevate the risk of infectious disease transmission through crowding and population movement. Although the World Health Organization recommends pre-event risk assessment, Korea previously lacked a standardized framework.

**② What new information is presented?**

The Korea Disease Control and Prevention Agency developed a standardized infectious disease risk assessment framework for mass gatherings. Application of the framework to two events demonstrated that repeated risk assessments identified preparedness gaps, informed mitigation measures, and reduced the final overall risk level.

**③ What are implications?**

The framework provides a basis for standardized preparedness planning and should be further refined and expanded for diverse mass gathering events.

## Introduction

A mass gathering event refers to an event where a large number of people with a specific purpose gather at a designated time and place, which can place a burden on the health response capacity of the host location [1]. International sports competitions, religious events, and cultural festivals fall into this category, and the risk of the occurrence and spread of infectious diseases can increase due to high density and contact among numerous participants, shared use of food and drinking water, and increased domestic and international population movement. Particularly for international mass gathering events, it is important to systematically assess risk factors of infectious disease and develop preparedness and response strategies before hosting the event, considering the potential importation of infectious diseases from abroad, transmission within the venue, and community spread.

The World Health Organization (WHO) developed the Generic All-Hazards Risk Assessment Tool for Mass Gathering Events [2] to systematically assess the health risks of mass gathering events. This tool comprehensively considers various hazards associated with events, including infectious diseases, environmental hazards, safety, and security, to assess risks and establish preparedness and response plans. In addition, countries and institutions have developed and operated their own tools to assess the risk factors and response capabilities of mass gathering events, such as Saudi Arabia’s all-hazards response tool, the Jeddah Tool [3], and the UK local government’s event-specific risk level calculation tool [4]. Although these tools differ in their assessment areas and operational methods, they share the commonality of determining risk levels based on assessment results and supporting the establishment of prevention and response plans.

Domestically, infectious disease preparedness and response procedures for each stage of an event and the roles of respective agencies have been refined through the “Standard Operating Procedure for Infectious Disease Preparedness and Response at Mass Gathering Events” [5]. However, there has been no standardized risk assessment framework capable of calculating an overall risk level by comprehensively scoring the infectious disease risk factors and preparedness and mitigation levels for each event and determining response directions based on those results.

Accordingly, the Korea Disease Control and Prevention Agency (KDCA) compared and analyzed the WHO and major foreign mass gathering event risk assessment frameworks through a 2024 policy research project and developed a Korean infectious disease risk assessment framework for mass gatherings that reflects the domestic infectious disease response system [6]. In addition, the research results were standardized into a mass gathering infectious disease risk assessment manual and an Excel form, and a foundation for field application was established for local governments through education and technical support. Since 2025, this framework has been applied to domestic mass gathering events to perform mass gathering event risk assessments, and the assessment results have begun to be utilized for establishing infectious disease preparedness and response plans and implementing mitigation measures.

This report aims to introduce the development process and main components of the Korean infectious disease risk assessment framework for mass gatherings and describe the results of field application of the risk assessment framework through domestic mass gathering event application cases. Furthermore, by presenting the changes in risk factors, mitigation factors, and overall risk levels identified through the repeated risk assessment process, this report aims to propose the feasibility of utilizing the standardized risk assessment framework for infectious disease preparedness and response plans for domestic mass gathering, along with directions for future supplementation.

## Methods

### 1. Subject of Analysis

This report introduces the development process and major components of the infectious disease risk assessment framework for mass gatherings developed by the KDCA and describes the implementation process and results of cases applying this framework to domestic mass gathering events. The application cases were selected from the 2025 domestic mass gathering event infectious disease risk assessment cases in which repeated assessments were conducted two or more times during the event preparation stage, allowing changes in risk factors, mitigation factors, and overall risk levels to be identified for each round. The cases included the 2025 Asian Artistic Gymnastics Championships held in Jecheon-si, Chungcheongbuk-do, and the 2025 Gwangju World Archery Championships held in Gwangju Metropolitan City were judged to be suitable for explaining the field application process of the risk assessment framework as their repeated assessment results and mitigation measure supplementation processes were documented. However, due to the nature of case reports, there are limitations in generalizing the research results to all mass gathering events. Furthermore, the risk assessment framework analyzed in this report is a risk assessment framework for infectious diseases developed to support the KDCA’s preparedness and response tasks for infectious diseases associated with mass gathering events. The scope of analysis was limited to infectious disease risk assessment among various hazards associated with mass gathering events.

### 2. Data Collection

To analyze the development process and application cases of the risk assessment framework, relevant documents were collected. For the analysis of the framework development process, the final report of the policy research service project, mass gathering event infectious disease risk assessment manual, and Excel form for risk assessment were utilized. For the analysis of application cases, the report on best practice cases of mass gathering event infectious disease risk assessment was used. The relevant data are administrative data from local governments and Regional centers for disease control and prevention, and this report focuses on describing the documented assessment results and mitigation measures.

### 3. Data Analysis

Based on the collected data, this study described the development process, configuration of assessment indicators, scoring methods, risk level calculation procedures, and the field dissemination process of the infectious disease risk assessment framework for mass gatherings. The application cases were summarized focusing on the event scale, participant characteristics, venue type, timing of assessment, risk factor scores, mitigation factor scores, overall risk level, and major supplementary measures. In cases of repeated risk assessments, scores for each round, changes in the overall risk level, and major mitigation measures were compared.

## Results

### 1. Development of a Mass Gathering Event Infectious Disease Risk Assessment Tool

In a policy research service project conducted by the KDCA to develop a mass gathering event risk assessment tool, six mass gathering event risk assessment tools currently in use by entities such as the WHO, Saudi Arabia, Canada, and the United Kingdom were reviewed, and a total of 422 assessment indicators were collected. Among the collected indicators, response capabilities that had low relevance to infectious disease transmission or were difficult to assess at the domestic event level, as well as redundant and similar items, were excluded or integrated.

The remaining indicators were classified into 13 risk factors and 12 mitigation factors to derive a draft of assessment indicators.

For the derived draft indicators, two rounds of Delphi surveys were conducted with 10 experts, including those in infectious diseases, preventive medicine, basic medicine, and clinical medicine, to review the importance, field measurability, and assessment relevance of the indicators; the final assessment indicators were selected by comprehensively considering content validity and priorities.

Moreover, based on the priorities derived from the Delphi surveys, weights reflecting the relative importance of each indicator were assigned to establish a risk level calculation system. Differential weights were applied to 6 risk factor indicators (0.408–0.028) and 7 mitigation factor indicators (0.228–0.020), and the sum of the weights for each domain was set to 1.

Finally, an assessment framework was established to calculate the overall risk level based on the scores of risk factors and mitigation factors. Additionally, to improve the consistency and utility of the assessment, an Excel form for risk assessment and a manual including assessment criteria and methods for each indicator and procedures for utilizing the results were prepared and distributed.

### 2. Composition of the Korean Infectious Disease Risk Assessment Framework for Mass Gatherings

#### 1) Basic direction and scope of application

The Korean mass gathering event infectious disease risk assessment is designed to assess the infectious disease risk factors and levels of preparedness and mitigation of mass gathering events that are planned in advance and held domestically under normal circumstances rather than national emergencies such as pandemics.

In principle, risk assessments are performed by the local governments with jurisdiction over the area hosting the event, whereas national-scale events that require a response across two or more regions are led by the KDCA.

Risk assessment is designed such that, in principle, the first assessment is conducted approximately 3 months before the event and repeated thereafter to reflect the status of event preparations and latest information obtained.

The assessment results were to be utilized not only to calculate the overall risk level but also as supporting data to identify vulnerabilities in infectious disease preparedness and response and to suggest response directions.

#### 2) Assessment indicators and risk level calculation

The assessment indicators consisted of risk factors and mitigation factors (Table 1). The risk factors comprised 6 indicators and 10 sub-indicators: the infectious disease situation in the countries of origin and residence of international participants, whether the event is held in multiple locations, venue type, ventilation, and crowd density, event duration, the level of compliance with prevention guidelines, and collective vocalization and physical activity.

The mitigation factors consisted of following indicators: infectious disease education and protective measures for event staff, provision of infectious disease information and emergency response guidance for attendees, personal hygiene education for attendees, indoor ventilation and air quality management, management of hygiene facilities, preparation of infectious disease response plans and procedures, and establishment of a risk communication system. In addition, there were 18 sub-indicators.

Each assessment indicator was scored based on a maximum of 10 points. Indicators consisting of a single item were assigned 0 or 10 points depending on whether the condition was met, and for indicators consisting of multiple sub-indicators, the assessment results of each sub-indicator were summed, ensuring that the total score for the corresponding item did not exceed 10 points.

In cases where objective information required for assessment is insufficient, to prevent underestimation, risk factors were to be assessed in the direction of high risk and mitigation factors in the direction of a low preparedness level. Additionally, in cases where the basis for assessment is insufficient, the final score is to be determined through discussions with experts from various fields, and the grounds for it must be sufficiently provided.

The calculated risk factor scores and mitigation factor scores were each classified into five levels: very low, low, moderate, high, and very high. They were then applied to a 5×5 decision matrix to determine the final overall risk level (Figure 1).

#### 3) Repeated risk assessment

The risk assessment was designed to be performed repeatedly by reflecting the latest information and preparedness and response plans secured during the event preparation process. By comparing the changes in the scores of risk factors and mitigation factors, as well as the overall risk level, for each round, it was made possible to identify areas requiring additional improvement.

#### 4) Utilization tool

To enhance the consistency and usability of the assessment, an Excel form for mass gathering event infectious disease risk assessment was developed. The form was configured so that, when assessment results by indicator (Yes/No/Insufficient Information) are entered, the scores for risk factors, mitigation factors, and the overall risk level are automatically generated. Furthermore, a mass gathering event infectious disease risk assessment manual presenting an overview of the risk assessment, its procedures, and assessment criteria for each indicator was developed and distributed to relevant organizations, such as local governments [7].

### 3. Field Dissemination of the Infectious Disease Risk Assessment Framework for Mass Gatherings

The KDCA conducted training and provided technical support for local governments to ensure that the developed infectious disease risk assessment framework for mass gatherings can be reliably used in the field. The training focused on introducing the basic concepts of risk assessment, assessment indicators and risk level calculation methods, how to use the Excel form for risk assessment, and practical application cases.

In 2025, training was conducted for 137 team leaders and staff in charge of infectious disease response at public health centers in 6 cities and provinces, and in 2026, training was conducted for 188 people in 7 cities and provinces as of July. In addition, in cooperation with Regional centers for disease control and prevention, technical support was provided to local governments preparing for actual mass gathering events.

These training and technical support activities constituted a process to support the use of the risk assessment framework developed through a policy research service project by local governments in the field. Through this, a foundation was laid for the central and local governments to utilize the same assessment indicators and procedures; however, in the future, it is necessary to monitor the actual assessment implementation rate and the quality of assessment results after training completion.

### 4. Application Cases

#### 1) 2025 Asian Artistic Gymnastics Championships (Jecheon-si, Chungcheongbuk-do)

The 2025 Asian Artistic Gymnastics Championships was held at the D Indoor Gymnasium from June 1 to 16, 2025. A total of 1,020 people participated, including 540 delegation members from 23 countries and 480 officials. The event risk assessment was conducted four times, starting from the event preparation stage, by the Infectious Disease Control Division of the Jecheon City Public Health Center and the Chungcheong Regional Center for Disease Control and Prevention.

In the first assessment, risk factors were 4.84 points (moderate) and mitigation factors were 0.98 points (very low), and the risk level was assessed as “high.” Thereafter, in the 2nd to 4th repeated assessments, major risk factors such as event scale, participating countries, and venue type remained unchanged, so the risk factor score was maintained at 4.84 points. On the contrary, as mitigation measures, such as establishing medical and response systems, training event staff, securing personal hygiene supplies, and inspecting the athletes’ village were gradually supplemented, the mitigation factor scores increased to 6.98 (high), 8.10 (very high), and 8.86 (very high). Accordingly, the risk level decreased to “low” in the second assessment and “very low” in the third and fourth assessments (Table 2).

In the initial assessment, some indicators requiring objective information were flagged for further verification by the responsible department, and thus were shared in advance with a request to reflect them in the safety management plan. Additionally, the operation of designated medical institutions, training for medical staff and event staff, management of hygiene facilities at stadiums and the athletes’ village, and the establishment of isolation spaces were identified as areas requiring additional verification and supplementation. Accordingly, infectious disease prevention and response training was conducted for medical staff and event staff, and personal hygiene guidelines such as cough etiquette, hand hygiene, and physical distancing were promoted to the participants. In addition, hand sanitizers and masks were secured and provided within the stadium medical room and the athletes’ village, an infectious disease emergency response team was organized, and response procedures in the event of an infectious disease outbreak and a contact system linking the event agency–designated medical institutions and public health centers, Regional Disease Response Centers, were established. In addition, the Chungcheong Regional Center for Disease Control and Prevention, the Infectious Disease Control Division and the Health and Hygiene Division of the Jecheon-si Public Health Center conducted a joint inspection of the athletes’ village and dining facilities, and carried out *Legionella* environmental testing on the athletes’ accommodations and stadiums.

In the Jecheon case, the major risk factor of being an indoor sports event with international participants was maintained during the competition period, but as insufficient indicators identified through repeated assessments were supplemented step-by-step, the mitigation factor score increased and the overall risk level in the final assessment was reduced from “high” to “very low.”

#### 2) 2025 Gwangju World Archery Championships (Gwangju Metropolitan City)

The 2025 Gwangju World Archery Championships were held from September 5 to 28, 2025, and approximately 1,200 people from 112 countries participated. The matches were held at Stadium A (preliminaries), Archery Field B (semifinals), and Plaza C (finals); because the environment and level of preparedness differed by venue, risk assessments were conducted separately for each venue. Stadium A and Archery Field B each underwent two risk assessments, whereas Plaza C, which was under construction at the time, underwent one assessment after completion.

In the first assessment, Stadium A had an overall risk level of “high,” with 7.59 points (high) for risk factors and 2.05 points (low) for mitigation factors. In the second assessment, although the risk factor score was raised to 8.88 points (very high), mitigation measures such as infectious disease education, checking the management status of hygiene facilities, and establishing a response system were supplemented, which increased the mitigation factor score to 10 points, and the final overall risk level was evaluated as “low.” Archery Field B also had a “very high” risk level in the first assessment, with a risk factor score of 8.88 (very high) and a mitigation factor score of 1.83 (very low); however, in the second assessment, the mitigation factor score rose to 10 points, decreasing the risk level to “low.” Plaza C had a risk factor score of 7.59 (high) and a mitigation factor score of 8.83 (very high), and the risk level was “low.” In the final assessment, the overall risk levels of all three venues were assessed as “low” (Table 3).

During the repeated assessment process, training and material distribution using the standard training plan for infectious disease prevention were conducted for event staff, and information on the infectious disease situation and emergency response was provided to participants. Additionally, a person in charge of ventilation and air purification facilities was designated to conduct inspections and replace filters, and additional sinks were secured within the facility. The preparedness plans for infectious disease outbreaks were established, and response and risk communication systems were established by operating city and district epidemic prevention task forces and a daily situation reporting system.

The Gwangju case is an example of individually assessing multiple venues to identify differences in risk factors and mitigation factors by location, and addressing deficiencies of each facility. Stadiums A and B, which underwent two rounds of assessment, showed a risk level of “high” or higher in the initial assessment; however, the overall risk level of all venues decreased as a result of implementing mitigation measures such as education, ventilation and facility management, expansion of hygiene facilities, and establishment of response systems through repeated assessments.

### 5. Comprehensive Interpretation of Application Cases

Both the Asian Artistic Gymnastics Championships in Jecheon and the Gwangju World Archery Championships conducted risk assessments during the event preparation stage and continuously supplemented the infectious disease preparedness and response systems based on the assessment results. In both cases, there were no significant changes in the risk factors of the events themselves during the assessment period; however, with the step-by-step implementation of mitigation measures—such as training for event staff, establishing medical and response systems, hygiene and ventilation management, and preparing response plans—the mitigation factor scores increased and the overall risk levels decreased to “low” or below.

The Jecheon case was an example in which repeated risk assessments were conducted four times for the same stadium, making it possible to repeatedly confirm changes in risk level according to improvements in the preparedness status. The Gwangju case is interpreted as an example demonstrating that even when operating plans and preparedness statuses differ by venue, individual risk assessments can be conducted for each venue to identify differences in risk factors and preparedness levels by location, apply appropriate improvement measures, and apply the same assessment framework to compare and inspect vulnerabilities and preparedness levels by location.

Meanwhile, the mass gathering event infectious disease risk assessment tool presented in this report is an assessment framework applied to mass gathering events held domestically. On the contrary, the infectious disease risk assessment for overseas mass gathering events conducted under the supervision of the KDCA is carried out based on the risk assessment methodology [8] of the European Centre for Disease Prevention and Control and assesses the exposure of domestic participants and the probability and impact after introduction into the country, considering the infectious disease situation in the host country. In other words, the KDCA separately applies risk assessment frameworks appropriate to the assessment purpose and scope of application for events held domestically and those held overseas.

### 6. Necessary Supplements for Mass Gathering Risk Assessment Tool Identified through Field Application

During the application process in the two cases, operational areas of the risk assessment tool requiring supplementation were also identified. First, for mass gathering events using multiple venues, it was necessary to more clearly specify the criteria for determining whether the assessment unit should be the entire event or individual venues. In the Gwangju case, each venue was independently assessed because the venue environments and preparedness levels differed by venue; however, in cases with multiple venues, it is necessary to further specify the criteria for setting the assessment unit and the method for interpreting the assessment results by location.

Second, in the early stages of event preparation, there were difficulties in assessment because objective information on some assessment indicators was not sufficiently secured, and the assessment results were sometimes changed later as new information was confirmed. In the Jecheon case, indicators requiring additional confirmation were identified in the initial assessment, and at Gwangju Stadium A, the risk factor score changed from 7.59 points to 8.88 points during the repeated assessment process. Therefore, during repeated assessments, there is a need to supplement the assessment procedures to record the assessment basis for each round and whether there is a lack of information and to ensure that the reasons are clearly documented if scores change.

Third, since this tool is utilized by various assessors, such as local governments, it is necessary to ensure consistency in indicator interpretation and scoring. Currently, conducting assessments is supported through training and technical support, but there is a need to review inter-assessor agreement and the reproducibility of repeated assessments using actual assessment data in the future, and based on the results, supplement the judgment criteria and cases for each indicator.

## Discussion

This report describes the development and standardization process of the mass gathering event infectious disease risk assessment tool developed through a policy research service project and presents its potential for field application in the infectious disease preparedness and response process for domestic mass gathering events through actual application cases. Through this, a standardized operational system was established to enable the central government and local governments to assess the infectious disease risk of mass gathering events according to the same assessment indicators and procedures, laying the foundation for application to various mass gathering events in the future.

In the two cases presented, repeated risk assessments were conducted from the event preparation stage and infectious disease preparedness and response measures were progressively supplemented based on the assessment results. This suggests that repeated risk assessments can be utilized to identify vulnerabilities and prioritize mitigation measures during the event preparation process. In particular, using the assessment results in consultations among local governments, Regional centers for disease control and prevention, and event organizers can support decision-making to refine infectious disease preparedness and response plans. Meanwhile, during the field application process, establishing assessment units for multiple venues, recording the basis for assessment and reasons for score changes during repeated assessments, and ensuring consistency in indicator interpretation and scoring among assessors were identified as areas for improvement. In the future, it is necessary to refine the assessment units and judgment criteria for each indicator based on actual assessment data and to examine inter-assessor agreement and the reproducibility of repeated assessments.

This report had several limitations. First, this risk assessment framework was developed focusing on mass gathering events held domestically; for mass gathering events held outside the country, there are limitations to its application as securing information on the host country’s infectious disease situation, healthcare system, and event operation may be limited. Second, because the analysis was limited to infectious disease risk assessment among the various hazards of mass gathering events, linkage with non-infectious disease hazards such as environmental hazards, safety, and security was not reviewed. Third, as the application cases accumulated to date are limited, the relationship between the risk assessment results and the actual occurrence of infectious diseases or risk reduction effects has not been sufficiently examined. In addition, the overall risk level is a relative risk level calculated by the assessment tool and does not directly indicate the actual likelihood of infectious disease occurrence or preventive effects. In the future, it is necessary to continuously accumulate assessment cases and analyze the status of infectious disease occurrence during and after events, the implementation of response measures, and inter-assessor agreement.

Infectious disease occurrence patterns and the international public health environment are continuously changing, and new risk factors, such as imported infectious diseases, emerging infectious diseases, and climate change-related infectious diseases, continue to emerge. Therefore, in the future, it is necessary to accumulate application cases for various types of mass gathering events while periodically re-evaluating and improving assessment indicators and weights to reflect new risk factors. Through such continuous supplementation, the infectious disease risk assessment framework for mass gatherings is expected to be used more reliably as the standard risk assessment framework for infectious disease preparedness and response for domestic mass gathering events.

## Figures and Tables

**Figure 1:**
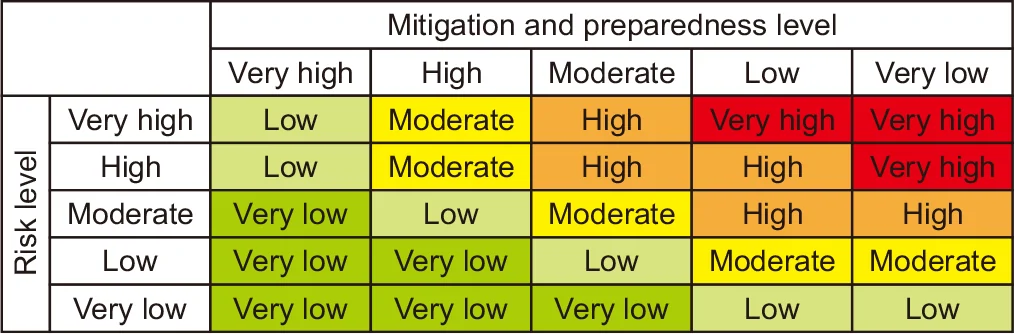
Matrix for determining the overall infectious disease risk level of mass gatherings Very low: 0≤x≤2, Low: 2<x≤4, Moderate: 4<x≤6, High: 6<x<8, Very high: 8≤x≤10.

**Table 1:** Indicators for infectious disease risk assessment at mass gatherings

Category	No.	Indicator	Sub-indicators	Scoring	Weight
Risk domain	1	Infectious disease situation in countries of origin and residence of international attendees	Presence of international attendees	Yes (10) No (0)	0.408
			Reports of infectious diseases with potential for international spread in attendees’ countries of origin or residence	Yes (10) No (0)	
	2	Multiple event venues	Whether the event is conducted at multiple venues (locations/regions)	Yes (10) No (0)	0.158
	3	Venue characteristics (indoor/outdoor setting, ventilation, and crowd density)	Venue type	Indoor (10) Indoor/Outdoor (10) Outdoor (0)	0.242
			Ventilation quality of indoor venues	Not applicable (outdoor) (0) Windows on front and side walls (3) Windows on one side only (6) No windows (10)	
			Crowd density (% of venue capacity)	≤24% (0) 25–49% (3) 50–74% (6) ≥75% (10)	
	4	Event duration	Duration of the event	1 day (2.5) 2–3 days (5) 4–6 days (7.5) ≥7 days (10)	0.061
	5	Expected adherence to infection prevention measures	Expected adherence to hand hygiene, use of hand sanitizer, respiratory etiquette, etc.	Low (10) Moderate (5) High (0)	0.103
	6	Group vocalization and physical activity	Participation in group vocalization (singing, cheering, chanting, etc.)	Yes (10) No (0)	0.028
			Participation in physically active programs (marching, dancing, games, etc.)	Yes (10) No (0)	
Mitigation domain	1	Infectious disease education and protective measures for event staff	Infectious disease education for medical personnel	Yes (10) No (0)	0.228
			Infectious disease education for operational staff		
			Availability of personal protective and hygiene supplies (e.g., hand sanitizer, face masks)		
	2	Provision of infectious disease information and emergency response guidance for attendees	Provision of information on circulating infectious diseases, their clinical characteristics, and recommended response measures before the event	Yes (10) No (0)	0.156
			Establishment of a plan for communicating information on infectious disease events occurring during or after the event		
			Provision of information on the location of on-site medical support teams		
	3	Personal hygiene education for attendees	Education or promotion on personal hygiene practices (e.g., respiratory etiquette, hand hygiene, and physical distancing)	Yes (10) No (0)	0.370
	4	Indoor ventilation and air quality management	Designation of ventilation personnel and training on indoor air quality management	Yes (10) No (0)	0.073
			Inspection of filters for ventilation and air purification systems		
			Establishment of a ventilation plan		
	5	Management of hygiene facilities	Availability and adequacy of handwashing facilities	Yes (10) No (0)	0.044
			Designation of cleaning personnel and establishment of a hygiene inspection plan		
	6	Preparation of infectious disease response plans and procedures	Deployment of medical personnel and medical support facilities	Yes (10) No (0)	0.109
			Patient transport system		
			Infectious disease response procedures		
	7	Risk communication system	Media communication plan	Yes (10) No (0)	0.020
			Infectious disease information-sharing system		
			Media support plan		

**Table 2:** Results of infectious disease risk assessments for the 2025 Asian Artistic Gymnastics Championships, Jecheon

Round	Assessment date	Risk level (score)	Mitigation level (score)	Overall risk level
1	March 10, 2025	Moderate (4.84)	Very low (0.98)	High
2	May 9, 2025	Moderate (4.84)	High (6.98)	Low
3	May 28, 2025	Moderate (4.84)	Very high (8.10)	Very low
4	June 5, 2025	Moderate (4.84)	Very high (8.86)	Very low

**Table 3:** Results of infectious disease risk assessments for the 2025 World Archery Championships, Gwangju

Venue	Round	Assessment date	Risk level (score)	Mitigation level (score)	Overall risk level
Venue A (preliminary round)	1	August 6, 2025	High (7.59)	Low (2.05)	High
	2	August 29, 2025	Very high (8.88)	Very high (10.00)	Low
Venue B (semifinal venue)	1	August 6, 2025	Very high (8.88)	Very low (1.83)	Very high
	2	August 29, 2025	Very high (8.88)	Very high (10.00)	Low
Venue C (final venue)	1	August 29, 2025	High (7.59)	Very high (8.83)	Low
