# Supplementary material for: Development and Application of an Infectious Disease Risk Assessment Framework for Mass Gatherings in the Republic of Korea
Source: Public Health Wkly Rep. 2026 Oct 22;19(40):1712–34. doi: 10.56786/PHWR.2026.19.40.3 (PMC13691638; doi:10.56786/PHWR.2026.19.40.3)
Supplement: Translation

# Translation: 군중모임행사 감염병 위험평가 체계 구축 및 국내 적용 사례

**Authors:** 정현 오, 신영 박, 영수 엄, 혜정 강, 무술 오, 정수 문, 젬마 박, 은영 김, 성순 김

**Affiliations:** 1 질병관리청 질병데이터과학분석관 역학데이터분석담당관; 2 제천시보건소; 3 전남광주통합특별시청; 4 질병관리청 충청권질병대응센터 감염병대응과; 5 질병관리청 호남권질병대응센터 감염병대응과

**Keywords:** 군중모임행사, 감염병 위험평가, 감염병 위험평가 도구, 감염병 관리

## Abstract

**목적:**

본 보고서는 질병관리청 군중모임행사 감염병 위험평가 체계의 구축 과정과 위험평가 도구의 주요 구성 요소를 기술하고, 국내 군중모임행사 적용 사례를 통해 현장 활용 가능성과 개선 필요사항을 분석하고자 하였다.

**방법:**

정책연구용역사업 결과보고서, 위험평가 매뉴얼 및 군중모임행사 위험평가 우수사례 보고서를 활용하여 위험평가 체계의 개발 과정과 구성 요소, 적용 결과를 분석하였다.

**결과:**

질병관리청은 2024년 정책연구용역사업을 통해 군중모임행사 감염병 위험평가 도구를 개발하였다. 이후 군중모임행사 감염병 대비ㆍ대응 표준운영절차와 연계하여 수행 절차와 주체 등을 포함한 한국형 군중모임행사 감염병 위험평가 체계를 구축하였다. 2025년부터 국내 군중모임행사에 해당 체계를 적용하였으며, 이 중 반복 위험평가가 수행된 2개 사례를 분석하였다. 두 사례 모두 초기 평가에서 확인된 미흡사항을 바탕으로 교육, 위생ㆍ환기 관리, 의료ㆍ대응체계 및 위험소통 체계를 보완하였고, 최종 평가에서 종합위험도는 모두 ‘낮음’ 이하로 산출되었다.

**결론:**

한국형 군중모임행사 감염병 위험평가 체계는 국내 군중모임행사의 감염병 위험요인과 대비ㆍ완화 수준을 표준화된 방식으로 점검하고, 행사 준비단계에서 개선 필요사항을 도출하는 데 활용될 수 있음을 보여주었다. 향후 다양한 행사 유형에 대한 적용 사례를 축적하고, 평가 결과와 실제 감염병 발생 또는 대응성과 간의 관련성을 검토할 필요가 있다.

**핵심요약**

**① 이전에 알려진 내용은?**

군중모임행사는 밀집 환경과 국내외 인구 이동으로 감염병 발생 및 확산 위험을 높일 수 있다. 세계보건기구 등은 행사 전 위험평가와 대비ㆍ대응계획 수립을 권고해 왔으나, 국내에는 이를 위한 표준화된 위험평가 도구가 부재하였다.

**② 새로이 알게 된 내용은?**

질병관리청은 2024년 정책연구용역사업을 통해 군중모임행사 감염병 위험평가 도구와 엑셀 기반 위험평가 서식을 개발하고, 이후 위험평가 매뉴얼을 작성ㆍ배포하였다. 우수사례 2건에서는 반복 위험평가를 통해 취약점을 확인하고, 그 결과를 바탕으로 교육, 위생ㆍ환기 관리, 대응체계 구축 등 완화조치를 보완함에 따라 최종 평가 위험도가 낮아졌다.

**③ 시사점은?**

본 체계는 군중모임행사의 감염병 대비ㆍ대응 수준을 체계적으로 점검ㆍ개선하는 표준화된 위험평가 체계로 활용될 수 있으며, 향후 다양한 행사 유형으로 확대ㆍ발전시킬 필요가 있다.

## 서 론

군중모임행사(Mass gathering)는 일정한 시간과 장소에 특정한 목적을 가진 다수의 사람이 모이는 행사로, 개최지의 보건 대응 역량에 부담을 줄 수 있는 행사를 의미한다[1]. 국제 스포츠대회, 종교행사, 문화축제 등이 이에 해당하며, 다수의 참가자가 밀집하여 접촉하고 식품ㆍ식수를 공동 이용하며 국내외 인구 이동이 증가하는 특성으로 인해 감염병의 발생 및 전파 위험이 높아질 수 있다. 특히 국제 군중모임행사는 해외 감염병의 유입, 행사장 내 전파 및 지역사회 확산 가능성을 고려하여 행사 개최 전 감염병 위험요인을 체계적으로 평가하고 대비ㆍ대응 전략을 마련하는 것이 중요하다.

세계보건기구(World Health Organization, WHO)는 군중모임행사의 보건위험을 체계적으로 평가하기 위해 Generic All-Hazards Risk Assessment Tool for Mass Gathering Events [2]를 개발하였다. 이 도구는 감염병뿐 아니라 환경적 위해, 안전ㆍ보안 등 행사와 관련된 다양한 위해요인을 종합적으로 고려하여 위험을 평가하고, 그 결과를 바탕으로 대비 및 대응계획을 수립하도록 하고 있다. 또한 이외에도 사우디아라비아의 전 위해요인(All-Hazards) 대응 도구인 Jeddah Tool [3], 영국 지방정부의 행사별 위험도 산출 도구[4] 등 각국 및 기관 단위에서도 군중모임행사의 위험요인과 대응역량을 평가하기 위한 자체 도구를 개발 및 운영해 왔다. 이들 도구는 평가영역과 운영 방법에는 차이가 있으나, 평가 결과를 바탕으로 위험수준을 결정하고 예방ㆍ대응계획 수립을 지원한다는 공통점을 가진다.

국내에서는 「군중모임행사 감염병 대비ㆍ대응 표준운영절차(Standard Operating Procedure)」 [5]를 통해 행사 단계별 감염병 대비ㆍ대응 절차와 기관별 역할을 정비해 왔다. 그러나 행사별 감염병 위험요인과 대비ㆍ완화 수준을 종합적으로 점수화하여 종합위험도를 산출하고, 그 결과를 근거로 대응방향을 결정할 수 있는 표준화된 위험평가 체계는 마련되어 있지 않았다.

이에 질병관리청은 2024년 정책연구용역사업을 통해 WHO 및 국외 주요 군중모임행사 위험평가 체계를 비교ㆍ분석하고, 국내 감염병 대응체계를 반영한 한국형 군중모임행사 감염병 위험평가 체계를 개발하였다[6]. 또한 연구 결과를 군중모임행사 감염병 위험평가 매뉴얼과 엑셀 기반 서식으로 표준화하고, 지방자치단체 교육과 기술지원을 통해 현장 활용 기반을 마련하였다. 2025년부터는 국내 군중모임행사에 이를 적용하여 군중모임행사 위험평가를 수행하고, 평가 결과를 감염병 대비ㆍ대응계획 수립과 완화조치 시행에 활용하기 시작하였다.

본 보고서는 한국형 군중모임행사 감염병 위험평가 체계의 구축 과정과 위험평가의 주요 구성 요소를 소개하고, 국내 군중모임행사 적용 사례를 통해 위험평가 체계의 현장 적용 결과를 기술하고자 하였다. 또한 반복 위험평가 과정에서 확인된 위험요인, 완화요인 및 종합위험도의 변화를 제시함으로써 국내 군중모임행사 감염병 대비ㆍ대응을 위한 표준 위험평가 체계의 활용 가능성과 향후 보완 방향을 제안하고자 하였다.

## 방 법

### 1. 분석대상

본 보고서는 질병관리청에서 개발한 군중모임행사 감염병 위험평가 체계의 구축 과정과 주요 구성 요소를 소개하고, 이를 국내 군중모임행사에 적용한 사례의 수행 과정과 결과를 기술하였다. 적용 사례는 2025년 국내 군중모임행사 감염병 위험평가 사례 중 행사 준비단계에서 2회 이상 반복 평가가 수행되어 회차별 위험요인, 완화요인 및 종합위험도의 변화를 확인할 수 있었던 사례를 선정하였다. 충청북도 제천시에서 개최된 2025 기계체조 아시아선수권대회와 광주광역시에서 개최된 2025 광주 세계양궁선수권대회를 포함하였으며, 두 사례는 반복 평가 결과와 완화조치 보완 과정이 문서화되어 있어 위험평가 체계의 현장 적용 과정을 설명하기에 적합한 사례로 판단하였다. 다만 사례보고의 특성상 연구 결과를 모든 군중모임행사에 일반화하는 데에는 제한이 있다. 또한 본 보고서에서 분석한 위험평가 체계는 질병관리청의 군중모임행사 감염병 대비ㆍ대응 업무를 지원하기 위해 개발된 감염병 대상 위험평가 체계로, 분석 범위는 군중모임행사와 관련된 다양한 위해요인 중 감염병 위험평가로 한정하였다.

### 2. 자료수집

위험평가 체계의 개발 과정과 적용 사례를 분석하기 위해 관련 문서를 수집하였다. 체계 개발 과정의 분석에는 정책연구용역사업 결과보고서, 군중모임행사 감염병 위험평가 매뉴얼, 엑셀 기반 위험평가 서식 등을 활용하였다. 적용 사례의 분석에는 군중모임행사 감염병 위험평가 우수사례 결과보고서를 활용하였다. 해당 자료는 지자체와 권역별 질병대응센터의 행정자료이며, 본 보고서에서는 문서화된 평가 결과와 완화조치를 중심으로 기술하였다.

### 3. 자료분석

수집된 자료를 바탕으로 군중모임행사 감염병 위험평가 체계의 개발 과정, 평가지표 구성, 점수화 방식, 위험도 산정 절차 및 현장 확산 과정을 기술하였다. 적용 사례는 행사 규모, 참가자 특성, 행사장 유형, 평가 시기, 위험요인 점수, 완화요인 점수, 종합위험도 및 주요 보완조치를 중심으로 정리하였다. 반복 위험평가 사례에서는 회차별 점수와 종합위험도 변화, 주요 완화조치를 비교하였다.

## 결 과

### 1. 군중모임행사 감염병 위험평가 도구의 개발

군중모임행사 위험평가 도구의 개발을 위해 질병관리청이 수행한 정책연구용역사업에서는 WHO, 사우디아라비아, 캐나다, 영국 등에서 활용 중인 6개의 군중모임행사 위험평가 도구를 검토하여 총 422개의 평가지표를 수집하였다. 수집된 지표 중 감염병 전파와 관련성이 낮거나 국내 행사 수준에서 평가하기 어려운 국가 단위 대응역량, 중복ㆍ유사 항목을 제외ㆍ통합하였다.

이후 남은 지표를 위험요인과 완화요인으로 분류하여 위험요인 13개와 완화요인 12개로 구성된 초안 평가지표를 도출하였다.

도출된 초안 지표는 감염학, 예방의학, 기초의학, 임상 전문가 등 10명을 대상으로 두 차례의 델파이조사를 실시하여 지표의 중요도, 현장 측정 가능성 및 평가 관련성을 검토하였으며, 내용타당도와 우선순위를 종합적으로 고려하여 최종 평가지표를 선정하였다.

또한 델파이 조사를 통해 도출된 우선순위를 기반으로 각 지표의 상대적 중요도를 반영한 가중치를 부여하여 위험도 산정체계를 마련하였다. 위험요인은 6개 지표(0.408–0.028), 완화요인은 7개 지표(0.228–0.020)에 차등 가중치를 적용하였으며, 각 영역의 가중치 합은 1이 되도록 설정하였다.

최종적으로 위험요인과 완화요인의 점수를 기반으로 종합위험도를 산출하는 평가체계를 구축하였다. 또한 평가의 일관성과 활용성을 높이기 위해 엑셀 기반 위험평가 서식을 개발하고, 지표별 평가 기준과 방법, 결과 활용 절차를 포함한 매뉴얼을 작성ㆍ배포하였다.

### 2. 한국형 군중모임행사 감염병 위험평가 체계의 구성

#### 1) 기본 방향과 적용 범위

한국형 군중모임행사 감염병 위험평가는 팬데믹 등 국가적 비상상황이 아닌 일반적 상황에서, 국내에서 사전 계획되어 개최되는 군중모임행사의 감염병 위험요인과 대비ㆍ완화 수준을 평가하도록 설계하였다.

위험평가는 기본적으로 행사 개최지역을 관할하는 지자체가 수행하며, 2개 이상의 권역에서 대응이 필요한 전국 규모의 행사는 질병관리청이 주관하도록 하였다.

위험평가는 행사 개최 약 3개월 전에 1차 평가를 실시하는 것을 원칙으로 하며, 이후 행사 준비상황과 확보되는 최신 정보를 반영하여 반복 수행하도록 설계하였다.

평가 결과는 종합위험도 산출뿐 아니라 감염병 대비ㆍ대응의 취약점을 확인하고 대응방향을 제시하는 근거자료로 활용하도록 하였다.

#### 2) 평가지표와 위험도 산정

평가지표는 위험요인과 완화요인으로 구성하였다(표 1). 위험요인은 국외 참여자의 출신 국가 및 체류국의 감염병 발생 상황, 다수의 장소에서 행사 개최 여부, 행사장 유형ㆍ환기ㆍ밀집도, 행사 진행 기간, 예방수칙 준수 수준, 집단 발성 및 신체활동의 6개 지표 및 10개 세부지표로 구성하였다.

완화요인은 운영인력 등 대상 감염병 교육 및 보호 조치, 참여자 대상 감염병 현황 및 응급대처 정보 제공, 참여자 대상 개인위생 교육, 실내 환기/공기 청정 관리, 위생 시설 관리 상태, 감염병 대응 계획/절차 마련, 위험소통 체계 마련의 지표 및 18개 세부 지표로 구성하였다.

각 평가지표는 10점 만점 기준으로 점수화하였다. 단일 항목으로 구성된 지표는 조건 충족 여부에 따라 0점 또는 10점을 부여하였고, 복수의 세부지표로 구성된 지표는 세부지표별 평가 결과를 합산하되 해당 항목의 총점이 10점을 초과하지 않도록 하였다.

평가에 필요한 객관적 정보가 부족한 경우, 과소평가를 방지하기 위해 위험요인은 높은 위험 방향으로, 완화요인은 낮은 준비 수준 방향으로 평가하도록 하였다. 또한 평가 근거가 불충분한 경우에는 다양한 분야의 전문가와 논의를 통해 최종 점수를 결정하고 그 근거를 충분히 제시하도록 하였다.

산출된 위험요인 점수와 완화요인 점수는 각각 매우 낮음, 낮음, 보통, 높음, 매우 높음의 5단계로 구분한 뒤, 5×5 의사결정 매트릭스에 적용하여 최종 종합위험도를 산정하였다(그림 1).

#### 3) 반복 위험평가

위험평가는 행사 준비과정에서 확보되는 최신 정보와 대비ㆍ대응 계획을 반영하여 반복 수행하도록 설계하였다. 회차별 위험요인과 완화요인의 점수 변화 및 종합위험도를 비교함으로써 추가 개선이 필요한 사항을 확인할 수 있도록 하였다.

#### 4) 활용도구

평가의 일관성과 활용성을 높이기 위해 엑셀 기반 군중모임행사 감염병 위험평가 서식을 개발하였다. 서식은 지표별 평가 결과(예/아니오/정보 부족)를 입력하면 위험요인, 완화요인 및 종합위험도를 자동으로 산출하도록 구성하였다. 아울러 위험평가의 개요, 절차, 지표별 평가 기준 등을 제시한 군중모임행사 감염병 위험평가 매뉴얼을 개발하여 지방자치단체 등 관계기관에 배포하였다[7].

### 3. 군중모임행사 감염병 위험평가 체계의 현장 확산

질병관리청은 개발된 군중모임행사 감염병 위험평가 체계가 현장에서 안정적으로 활용될 수 있도록 지방자치단체를 대상으로 교육과 기술지원을 실시하였다. 교육은 위험평가의 기본 개념, 평가지표와 위험도 산정 방법, 엑셀 기반 위험평가 서식 활용법 및 실제 적용 사례 소개를 중심으로 운영하였다.

2025년에는 6개 시ㆍ도 보건소 감염병대응 팀장 및 실무자 137명을 대상으로 교육을 실시하였으며, 2026년에는 7월 기준으로 7개 시ㆍ도 188명을 대상으로 교육을 실시하였다. 또한 권역별 질병대응센터와 협력하여 실제 군중모임행사를 준비하는 지방자치단체에 기술지원을 실시하였다.

이러한 교육과 기술지원은 정책연구용역사업을 통해 개발된 위험평가 체계를 지자체 현장에서 활용하도록 지원하는 과정이었다. 이를 통해 중앙정부와 지방자치단체가 동일한 평가지표와 절차를 활용할 수 있는 기반을 마련하였으나, 향후에는 교육 이수 후 실제 평가 수행률과 평가 결과의 질을 함께 점검할 필요가 있다.

### 4. 적용 사례

#### 1) 2025 기계체조 아시아선수권대회(충청북도 제천시)

2025 기계체조 아시아선수권대회는 2025년 6월 1일부터 16일까지 D 실내체육관에서 개최되었으며, 23개국 선수단 540명과 관계자 480명 등 총 1,020명이 참가하였다. 대회 위험평가는 제천시보건소 감염병관리과와 충청권질병대응센터가 행사 준비단계부터 총 4회 수행하였다.

1차 평가에서는 위험요인은 4.84점(보통), 완화요인은 0.98점(매우 낮음)이었으며, 위험도는 ‘높음’으로 평가되었다. 이후 2–4차 반복 평가에서 행사 규모, 참가국, 행사장 유형 등 주요 위험요인은 변동이 없어 위험요인 점수는 4.84점으로 유지되었다. 반면 의료ㆍ대응체계 마련, 운영인력 교육, 개인위생 물품 확보, 선수촌 점검 등 완화조치가 단계적으로 보완되면서 완화요인 점수는 6.98점(높음), 8.10점(매우 높음), 8.86점(매우 높음)으로 상승하였다. 이에 따라 위험도는 2차 평가에서 ‘낮음’, 3차와 4차 평가에서 ‘매우 낮음’으로 감소하였다(표 2).

초기 평가에서는 객관적 정보 확인이 필요한 일부 지표는 주관부서의 추가 확인이 필요한 항목으로 판단되어 사전에 공유하고, 안전관리계획에 반영하도록 요청하였다. 또한 지정 의료기관 운영, 의료진과 운영인력 대상 교육, 경기장 및 선수촌의 위생시설 관리, 격리공간 마련 등이 추가 확인과 보완이 필요한 사항으로 확인되었다. 이에 의료진과 운영인력을 대상으로 감염병 예방ㆍ대응 교육을 실시하고, 대회 참가자에게 기침 예절, 손 위생, 신체적 거리 두기 등 개인위생 수칙을 홍보하였다. 또한 경기장 의무실과 선수촌 내에 손 소독제와 마스크를 확보ㆍ제공하고, 감염병 비상대책반 편성, 감염병 발생 시 대응 절차 및 행사 대행사-지정 의료기관-보건소-권역센터 간 연락체계를 구축하였다. 아울러 충청권질병대응센터 및 제천시보건소 감염병관리과, 보건위생과에서 선수촌과 식당을 합동 점검하고, 선수단 숙소와 경기장에 대한 레지오넬라 환경검사를 실시하였다.

제천 사례에서는 국제 참가자가 참여하는 실내 스포츠 행사라는 주요 위험요인이 대회 기간 동안 유지되었으나, 반복 평가를 통해 확인된 미흡한 지표를 단계적으로 보완하면서 완화요인 점수가 높아졌고, 최종 종합위험도는 ‘높음’에서 ‘매우 낮음’으로 낮아졌다.

#### 2) 2025 광주 세계양궁선수권대회(광주광역시)

2025 광주 세계양궁선수권대회는 2025년 9월 5일부터 28일까지 개최되었으며, 112개국에서 약 1,200명이 참가하였다. 경기는 A 경기장(예선), B 양궁장(준결승전), C 광장(결승)에서 진행되었으며 경기장별 환경과 준비 수준이 상이하여 각 경기장을 구분하여 위험평가를 수행하였다. A 경기장과 B 양궁장은 각각 두 차례 위험평가를 실시하였으며, 당시 공사가 진행 중이던 C 광장은 완공 이후 1회 평가를 실시하였다.

A 경기장은 1차 평가에서 위험요인 7.59점(높음), 완화요인 2.05점(낮음)으로 종합위험도가 ‘높음’이었다. 2차 평가에서는 위험요인 점수가 8.88점(매우 높음)으로 상향되었으나, 감염병 교육, 위생시설 관리 상태 점검 및 대응체계 마련 등 완화조치가 보완되어 완화요인 점수가 10점으로 상승하였고, 최종 종합위험도는 ‘낮음’으로 평가되었다. B 양궁장도 1차 평가에서는 위험요인 8.88점(매우 높음), 완화요인 1.83점(매우 낮음)으로 위험도가 ‘매우 높음’이었으나, 2차 평가에서 완화요인이 10점으로 상승하여 위험도가 ‘낮음’으로 감소하였다. C 광장은 위험요인 7.59점(높음), 완화요인 8.83점(매우 높음)으로 위험도는 ‘낮음’이었다. 최종 평가에서 세 경기장의 종합위험도는 모두 ‘낮음’으로 평가되었다(표 3).

반복 평가 과정에서는 운영인력 대상으로 감염병 예방 표준교육안을 통한 교육 및 자료 배포를 실시하고, 참여자에게 감염병 현황과 응급대처 정보를 제공하였다. 또한 환기ㆍ공기청정 시설 담당자를 지정하여 점검하고 필터를 교체하였으며, 시설 내 세면대를 추가로 확보하였다. 아울러 감염병 발생 대비 계획을 수립하고, 시ㆍ구 방역대책반과 일일상황보고체계를 운영하여 대응 및 위험소통 체계를 구축하였다.

광주 사례는 복수 경기장을 개별 평가하여 장소별 위험요인과 완화요인의 차이를 확인하고, 시설별 미흡사항을 보완한 사례이다. 2회의 평가를 시행한 A, B 경기장은 초기 평가에서 ‘높음’ 이상의 위험도를 나타냈으나, 반복 평가를 통해 교육, 환기ㆍ시설관리, 위생시설 확충 및 대응체계 마련 등의 완화조치를 이행한 결과 모든 경기장의 종합위험도가 감소하였다.

### 5. 적용 사례 종합 해석

제천 기계체조 아시아선수권대회와 광주 세계양궁선수권대회 모두 행사 준비단계에서 위험평가를 실시하고, 평가 결과를 바탕으로 감염병 대비ㆍ대응체계를 지속적으로 보완하였다. 두 사례 모두 행사 자체의 위험요인은 평가 기간 동안 큰 변화가 없었으나, 운영인력 교육, 의료 및 대응체계 구축, 위생ㆍ환기 관리, 대응계획 마련 등 완화조치가 단계적으로 이루어지면서 완화요인 점수가 증가하였고, 종합위험도는 모두 ‘낮음’ 이하 수준으로 감소하였다.

제천 사례는 동일한 경기장을 대상으로 4회 반복 위험평가를 수행하여 준비상황의 개선에 따른 위험도 변화를 반복적으로 확인할 수 있었던 사례였다. 광주 사례는 경기장별로 개별 위험평가를 실시하여 장소별 위험요인과 준비 수준의 차이를 확인하고 이에 맞는 개선조치를 적용하여 경기장별 운영계획과 준비상황이 상이한 경우에도 동일한 평가체계를 적용하여 장소별 취약점과 준비 수준을 비교ㆍ점검할 수 있음을 보여주는 사례로 해석된다.

한편, 본 보고서에서 제시한 군중모임행사 감염병 위험평가 도구는 국내에서 개최되는 군중모임행사를 대상으로 적용되는 평가체계이다. 반면 질병관리청이 주관하여 수행하는 국외 군중모임행사 감염병 위험평가는 유럽질병예방통제센터(European Centre for Disease Prevention and Control)의 위험평가 방법론[8]을 기반으로 수행하고 있으며, 개최국의 감염병 발생 상황을 고려하여 국내 참가자의 노출 및 국내 유입 후 발생 가능성과 영향력을 평가한다. 즉, 질병관리청은 국내 개최 행사와 국외 개최 행사의 평가 목적 및 활용 범위를 고려하여 위험평가 체계를 구분하여 적용하고 있다.

### 6. 현장 적용을 통해 확인된 군중모임행사 감염병 위험평가 도구 보완 필요사항

두 사례의 적용 과정에서는 위험평가 도구의 운영상 보완 필요사항도 확인되었다. 첫째, 복수의 행사장을 사용하는 군중모임행사에서는 행사 전체와 개별 행사장 중 어느 수준을 평가 단위로 설정할 것인지에 대한 기준을 보다 명확히 제시할 필요가 있었다. 광주 사례에서는 경기장마다 행사장 환경과 준비 수준에 차이가 있어 각 경기장을 독립적인 평가 단위로 설정하였으나, 복수 행사장을 사용하는 경우 평가 단위를 설정하는 기준과 장소별 평가 결과의 해석 방법을 보다 구체화할 필요가 있다.

둘째, 행사 준비 초기에는 일부 평가지표의 객관적 정보가 충분히 확보되지 않아 평가에 어려움이 있었으며, 이후 새로운 정보가 확인되면서 평가 결과가 변경되기도 하였다. 제천 사례에서는 초기 평가에서 추가 확인이 필요한 지표가 확인되었으며, 광주 A 경기장에서는 반복 평가 과정에서 위험요인 점수가 7.59점에서 8.88점으로 변경되었다. 따라서 반복 평가 시 회차별 평가 근거와 정보 부족 여부를 기록하고, 점수가 변경된 경우 그 사유를 명확히 문서화할 수 있도록 평가 절차를 보완할 필요가 있다.

셋째, 본 도구는 지방자치단체 등 다양한 평가자가 활용하므로 지표 해석과 점수 부여의 일관성을 확보할 필요가 있다. 현재 교육과 기술지원을 통해 평가 수행을 지원하고 있으나, 향후 실제 평가자료를 활용하여 평가자 간 일치도와 반복 평가의 재현성을 검토하고, 그 결과를 바탕으로 지표별 판단기준과 사례를 보완할 필요가 있다.

## 논 의

본 보고서는 정책연구용역사업을 통해 개발된 군중모임행사 감염병 위험평가 도구의 개발과 표준화 과정을 기술하고, 실제 적용 사례를 통해 국내 군중모임행사 감염병 대비ㆍ대응 과정에서의 현장 활용 가능성을 제시하였다. 이를 통해 중앙정부와 지방자치단체가 동일한 평가지표와 절차에 따라 군중모임행사의 감염병 위험을 평가할 수 있는 표준화된 운영체계를 구축하였으며, 향후 다양한 군중모임행사에 적용할 수 있는 기반을 마련하였다.

제시된 두 사례에서는 행사 준비단계부터 반복 위험평가를 수행하고, 평가 결과를 바탕으로 감염병 대비ㆍ대응 조치를 단계적으로 보완하였다. 이는 반복 위험평가가 행사 준비 과정에서 취약점을 확인하고 완화조치의 우선순위를 정하는 데 활용될 수 있음을 시사한다. 특히 평가 결과를 지자체, 권역질병대응센터 및 행사 주관기관 간 협의에 활용함으로써 감염병 대비ㆍ대응계획을 구체화하는 의사결정 지원도구로 기능할 수 있다. 한편, 현장 적용 과정에서는 복수 행사장의 평가 단위 설정, 반복 평가 시 평가 근거와 점수 변경 사유의 기록, 평가자 간 지표 해석과 점수 부여의 일관성 확보가 보완 과제로 확인되었다. 향후 실제 평가자료를 바탕으로 평가 단위와 지표별 판단기준을 구체화하고, 평가자 간 일치도와 반복 평가의 재현성을 검토할 필요가 있다.

본 보고서에는 몇 가지 제한점이 있다. 첫째, 본 위험평가 체계는 국내에서 개최되는 군중모임행사를 중심으로 개발되었으며, 국외 군중모임행사의 경우 개최국의 감염병 발생 상황, 의료체계 및 행사 운영 정보의 확보가 제한될 수 있어 적용에 한계가 있다. 둘째, 군중모임행사의 다양한 위해요인 중 감염병 위험평가에 한정하여 분석하였으므로 환경적 위해, 안전ㆍ보안 등 비감염병 위해요인과의 연계는 검토하지 못하였다. 셋째, 현재까지 축적된 적용 사례가 제한적이므로 위험평가 결과와 실제 감염병 발생 여부 또는 위험 감소 효과 간의 관련성을 충분히 검토하지 못하였다. 또한 종합위험도는 평가도구로 산출된 상대적 위험수준이며, 실제 감염병 발생 가능성이나 예방 효과를 직접 의미하지는 않는다. 향후에는 평가 사례를 지속적으로 축적하고, 행사 중ㆍ후 감염병 발생 현황, 대응조치 이행 여부, 평가자 간 일치도 등을 함께 분석할 필요가 있다.

감염병 발생 양상과 국제 공중보건 환경은 지속적으로 변화하고 있으며 해외유입 감염병, 신종감염병 및 기후변화와 관련된 감염병 등 새로운 위험요인이 계속 등장하고 있다. 따라서 향후에는 다양한 유형의 군중모임행사 적용 사례를 축적하는 한편, 새로운 위험요인을 반영하여 평가지표와 가중치를 주기적으로 재평가하고 개선할 필요가 있다. 이러한 지속적인 보완을 통해 군중모임행사 감염병 위험평가 체계는 국내 군중모임행사의 감염병 대비ㆍ대응을 위한 표준 위험평가 체계로 더욱 안정적으로 활용될 수 있을 것으로 기대된다.

## Figures and Tables

**그림 1:** 군중모임행사 감염병 종합위험도 산정을 위한 매트릭스 매우 낮음: 0≤x≤2, 낮음: 2<x≤4, 보통: 4<x≤6, 높음: 6<x<8, 매우 높음: 8≤x≤10.

**표 1:** 군중모임행사 감염병 위험평가 평가지표

구분	연번	평가지표	세부지표	점수	가중치
위험요인	1	국외 참여자의 출신 국가 및 체류국의 감염병 발생 상황	국외 참여자 포함 여부	예(10) 아니오(0)	0.408
			국외 참여자들의 출신/체류국에서 국제적 확산 위험이 있는 감염병 보고 여부	예(10) 아니오(0)	
	2	다수의 장소에서 행사 개최	다수의 장소에서 행사 개최 여부(장소, 지역)	예(10) 아니오(0)	0.158
	3	장소(실내/실외), 환기상태, 밀집도	행사장소 유형	실내(10) 실내·외(10) 실외(0)	0.242
			행사 장소에 실내가 포함되는 경우, 해당 장소의 환기 수준 정도	해당 없음(야외) (0) 전후면, 전측면 창문(3) 단측면 창문(6) 창문 없음(10)	
			장소의 밀집도(수용 가능 인원의 백분율)	≤24% (0) 25–49% (3) 50–74% (6) ≥75% (10)	
	4	행사 진행 기간	행사 진행 기간(구간)	1일(2.5) 2–3일(5) 4–6일(7.5) 7일 이상(10)	0.061
	5	예방 수칙 준수 수준	참여자 예방수칙 준수 예상 수준(손 씻기, 손 세정제 사용, 호흡기 예절 등)	낮음(10) 보통(5) 높음(0)	0.103
	6	집단 발성(노래, 환호 등) 및 기립/신체 활동	참여자 행사 중 집단 발성 참여 여부(노래 부르기, 환호, 구호 외치기 등)	예(10) 아니오(0)	0.028
			참여자 행사 중 활동성 프로그램 참여 여부 (행진, 춤추기, 게임 등)	예(10) 아니오(0)	
완화요인	1	운영인력 등 대상 감염병 교육 및 보호 조치	의료직원 대상 감염병 교육 여부	예(10) 아니오(0)	0.228
			운영인력 대상 감염병 교육 여부	예(10) 아니오(0)	
			개인위생 물품(손소독제, 마스크 등) 확보 및 제공 여부	예(10) 아니오(0)	
	2	참여자 대상 감염병 현황 및 응급대처 정보 제공	(행사 전) 현재 유행하는 감염병 종류, 임상적 특징, 대응방법 정보 제공 여부	예(10) 아니오(0)	0.156
			(행사 중, 후) 행사장 내 감염병 발생 시 정보 제공 계획 수립 여부	예(10) 아니오(0)	
			행사장 내 의료지원팀 위치 정보 제공 여부	예(10) 아니오(0)	
	3	참여자 대상 개인위생 교육	개인위생(기침 예절, 손 위생, 신체적 거리 두기 등) 교육 또는 홍보 여부	예(10) 아니오(0)	0.370
	4	실내 환기/공기 청정 관리	환기 담당자 지정 및 실내 공기질 관리 교육 여부	예(10) 아니오(0)	0.073
			공기질 관리 장치(환기팬, 에어컨 및 공기청정기 등) 필터 점검 여부	예(10) 아니오(0)	
			환기 계획 수립 여부	예(10) 아니오(0)	
	5	위생 시설 관리 상태	손 씻기 시설(화장실 내 세면대 등) 확보 및 충분 여부	예(10) 아니오(0)	0.044
			청소담당자 지정 및 위생 상태 점검 계획 수립 여부	예(10) 아니오(0)	
	6	감염병 대응 계획/절차 마련	의료인력 배치 및 의료 지원 시설 확보 여부	예(10) 아니오(0)	0.109
			감염병 발생 시 환자 이송 체계 마련 여부	예(10) 아니오(0)	
			감염병 발생 시 대응 절차 마련 여부	예(10) 아니오(0)	
	7	위험소통 체계 마련	감염병 발생 시 보도 계획 수립 여부	예(10) 아니오(0)	0.020
			감염병 관련 정보 공유 체계 확보 여부	예(10) 아니오(0)	
			감염병 발생 시 취재지원 계획 수립 여부	예(10) 아니오(0)	

**표 2:** 2025 기계체조 아시아선수권대회 군중모임행사 감염병 위험평가 결과

회차	날짜	위험요인(점수)	완화요인(점수)	종합위험도
1	2025.3.10.	보통(4.84)	매우 낮음(0.98)	높음
2	2025.5.9.	보통(4.84)	높음(6.98)	낮음
3	2025.5.28.	보통(4.84)	매우 높음(8.10)	매우 낮음
4	2025.6.5.	보통(4.84)	매우 높음(8.86)	매우 낮음

**표 3:** 2025 광주 세계양궁선수권대회 군중모임행사 감염병 위험평가 결과

장소	회차	날짜	위험요인(점수)	완화요인(점수)	종합위험도
A 경기장(예선)	1	2025.8.6.	높음(7.59)	낮음(2.05)	높음
	2	2025.8.29.	매우 높음(8.88)	매우 높음(10.00)	낮음
B 양궁장(준결승전)	1	2025.8.6.	매우 높음(8.88)	매우 낮음(1.83)	매우 높음
	2	2025.8.29.	매우 높음(8.88)	매우 높음(10.00)	낮음
C 광장(결승)	1	2025.8.29.	높음(7.59)	매우 높음(8.83)	낮음
